# Survival Benefits of Simple *Versus* Extended Cholecystectomy and Lymphadenectomy for Patients With T2 Gallbladder Cancer: A Propensity-Matched Population-Based Study (2010 to 2015)

**DOI:** 10.3389/fonc.2021.705299

**Published:** 2021-08-26

**Authors:** Wei Zhang, Zhangkan Huang, Wen-er Wang, Xu Che

**Affiliations:** ^1^Department of Hepatobiliary Surgery, People’s Hospital of Xiangxi Autonomous Prefecture, Jishou, China; ^2^Department of Hepatobiliary and Pancreatic Surgery, National Cancer Center/National Clinical Research Center for Cancer/Cancer Hospital & Shenzhen Hospital, Chinese Academy of Medical Sciences and Peking Union Medical College, Shenzhen, China; ^3^Department of Pancreatic and Gastric Surgery, National Cancer Center/National Clinical Research Center for Cancer/Cancer Hospital, Chinese Academy of Medical Sciences and Peking Union Medical College, Beijing, China

**Keywords:** cholecystectomy, lymphadenectomy, gallbladder cancer, surgical resection, surveillance

## Abstract

**Objective:**

This article aims to evaluate the survival benefits of simple cholecystectomy, extended cholecystectomy, as well as scope regional lymphadenectomy for T2 gallbladder cancer (GBC) patients.

**Methods:**

We identified eligible patients from the Surveillance, Epidemiology, and End Results database. The confounding factors were controlled *via* propensity score matching. The log-rank test was utilized to compare overall survival. The multivariate Cox regression was then used to determine risk factors.

**Results:**

Overall, data from 1,009 patients were obtained. The median overall survival (OS) of 915 patients that underwent simple cholecystectomy was 15 months; the median OS of 94 patients that underwent extended cholecystectomy was 17 months. There were no significant differences before and after propensity score matching (p = 0.542 and p = 0.258). The patients who received regional lymphadenectomy did show significant survival benefit, compared to those who did not receive regional lymphadenectomy. Furthermore, this benefit is observed in the N0 stage, but not observed in the N1 stage. In addition, the OS of patients who received lymphadenectomy for four or more regions was significantly better than those who received one to three regions lymphadenectomy. Age, the scope of regional lymphadenectomy, N stage, and tumor size were identified as prognostic factors.

**Conclusions:**

Extended cholecystectomy was not observed to significantly improve postoperative prognosis of patients with T2 GBC. However, there was a significant survival benefit shown for those with regional lymphadenectomy, particularly for patients with negative lymph nodes. Future studies on the control of potential confounding factors and longer follow-ups are still needed.

## Introduction

Gallbladder cancer (GBC) is one of the most common malignant tumors of the biliary system and accounts for about 46%. Although the incidence is not very high, it usually exhibits poor prognosis due to its high malignancy of biological characteristics, as well as insensitivity to radiotherapy or chemotherapy. The 5-year overall survival (OS) is only 5%, but the incidence is on the rise (1). Currently, the preferred treatment for GBC is surgery. T2 GBC is defined as invasion limited to the connective tissue surrounding the muscle layer of the gallbladder, but not breaking through the serosal layer or invading the liver. Compared to the 7th edition of “American Joint Committee on Cancer (AJCC) Tumor TNM Staging Manual”, the 8th edition subdivides the T2 stage into T2a stage (in which the tumor is on the peritoneal side) and T2b stage (in which the tumor is on the liver side). However, the scope of surgical resection for both T2a and T2b GBC remains controversial. In addition, the lymph node metastasis rate in T2 stage of GBC is 46% (2). If there are more than one positive lymph nodes, the lymph node staging is N1 or N2. Regional lymph node dissection can help improve postoperative prognosis. Any metastasis that involves tissues other than the lymph nodes is classified as the M1 stage.

Despite the fact that the National Comprehensive Cancer Network (NCCN) guidelines have recommended use of extended cholecystectomy for T1b–T4 GBC, these suggestions have only been based on a few retrospective studies (3–6), which tend to be less reliable. Many studies have shown that extended cholecystectomy does not improve postoperative survival (7, 8). The data from the Surveillance, Epidemiology, and End Results (SEER) database, released in 2018, demonstrate that extended resection and lymphadenectomy have not been fully implemented in the treatment of early stage GBC, including T2 GBC. The failure to follow the guidelines of GBC treatment may be due to controversy over the benefits of extended cholecystectomy *versus* simple cholecystectomy. Currently, there is still a lack of research on the SEER data of T2 GBC. Therefore, collecting treatment data from GBCs of the SEER data is very important for clinical instructions.

## Patients and Methods

### Source of Data and Inclusion Criteria

Herein, we evaluated the survival in patients with T2 GBC in the National Cancer Institute’s SEER program. The SEER database was initially established by the National Cancer Institute of the United States in 1973 for the National Institutes of Health (NIH) monitoring research project, with the purpose of collecting cancer statistical data in the United States. To date, the database has continuously collected data on cancer incidence, prevalence, and survival of patients from 18 regional registries and tracks approximately 34.6% of the United States population. In this population-based retrospective cohort study, we evaluated prognosis of patients that were diagnosed with primary T2 GBC whose clinical data were incorporated to the SEER database between 2010 and 2015.

GBC was defined according to the 3rd edition of the International Classification of Diseases for Oncology (ICD-O-3) (code C23.9). Histological types of GBC include adenocarcinoma (code 8140-8147), papillary or papillary adenocarcinoma (code 8050-8052 and 8260-8263), signet ring cell carcinoma (code 8490), small cell carcinoma (code 8040-8046), unspecified cancer (code 8010-8015), and undifferentiated cancer (code 8020-8022). Patients were excluded from the study if they had uncertain histology, incomplete or missing data, had suffered previous or secondary cancer, or lack information about any type of surgery. In the SEER database, TNM staging is based on 7th edition of the American Joint Committee on Cancer (AJCC), in which N staging is defined, according to site of lymphatic drainage. Lack of regional lymph node metastasis was defined as “N0”. The metastasis in lymph nodes around the cystic duct, common bile duct, hepatic artery, and portal vein was defined as “N1”. Metastasis in lymph nodes around the abdominal trunk, around the head of the pancreas, around the superior mesenteric artery, and around the abdominal aorta was defined as “N2”. Furthermore, the “CS Tumor Size/Ext Eval (2004+)” item of our screened patients is “3”, which is explained in the “CS Manual Online” as “pathological staging”.

Age, race, and gender were selected as demographic characteristics. Other baseline characteristics included primary site of the tumor, histological grade, AJCC N stage, tumor size, type of surgery, and regional lymphadenectomy (LN). It is important to note that in the SEER database, the histological grade of the tumors is divided into four, namely, Grade I (well-differentiated), Grade II (moderately differentiated), Grade III (poorly differentiated), and Grade IV (undifferentiated or anaplastic). We classified surgical status based on records in the SEER database. The “30 or 40” (NAACCR item 1290, codes 30 and 40) of the item “RX Summ–Surg Prim Site (1998+)” is interpreted as “simple cholecystectomy”. The “60” (NAACCR item 1290, codes 40) of the item “RX Summ–Surg Prim Site (1998+)” is interpreted as “extended cholecystectomy”. The cancer-specific survival (CSS) in this study is defined as duration from the date of diagnosis until death due to GBC or other causes. Herein, we calculate both GBC-specific survival (G-CSS) and Other-CSS (O-CSS). The SEER Cancer Registry utilizes certain algorithms to extract cause of death from the death certificate in order to determine a single, specific cause of death. In some cases, attribution of a single cause of death may be difficult, and erroneous attribution can occur. For example, death can be attributed to site of metastasis, rather than original site of the tumor. To obtain deaths related to the specific cancers that are not coded, the SEER cause-specific death classification variables were defined by considering the causes of deaths in conjunction with tumor sequence (*i.e.*, only one primary or one of multiple primaries), site of the original cancer diagnosis, as well as comorbidities (*e.g.*, AIDS and/or site-related diseases).

### Statistical Analysis

The categorical variables were summarized as frequencies (%), and compared using χ2 or the Fisher’s exact tests, as deemed appropriate. Continuous variables were presented as mean ± standard deviation or median [interquartile range (IQR)] and compared utilizing independent samples T-test or the Mann–Whitney U test. The survival curve was then determined using Kaplan–Meier methodology and compared using log-rank test. Hazard ratio (HR) was estimated through the use of the Cox proportional hazard regression model. SEER data were extracted by utilizing the Client-Server Mode with SEER*Stat 8.3.5. Statistical analyses were carried out by using SPSS Statistics version 24 (IBM, Chicago, IL), and R software and its optional packages. A two-tailed p-value <0.05 was considered to be statistically significant.

### Multivariate Regression Analysis and Nomogram Construction

Multivariate regression analysis was carried out using the Cox proportional hazard model. The interaction among multiple variables was analyzed *via* the Cox model in order to reflect their impact on survival benefit. The coefficients from multivariate Cox regression analysis were utilized for nomogram construction in order to predict the survival outcomes. Two dimensions of calibration and discrimination were utilized to validate the model performance by using the calibration curve and the receiver operating characteristic (ROC) curve, respectively.

### Propensity Score Matching

The outcomes of this study included OS and CSS. Patient characteristics, such as age, gender, race, N-stage, histological classification, surgical method, and regional lymphadenectomy, were collected. Due to uneven distribution of patient characteristics within the SEER database, the conclusions were found to be statistically biased. In order to adjust for this bias, propensity score matching (PSM) was proposed to control bias of covariates that affects the treatment selection process. Herein, we identified important prognostic factors including age, race, N-stage, scope of regional lymphadenectomy, in combination with available records from SEER, which were then used as covariates for propensity score modeling. The propensity score was defined as the probability of patients that underwent extended cholecystectomy or simple cholecystectomy, estimated *via* a non-parsimonious multivariable logistic regression model. The nearest neighbor matching algorithm (ratio = 1:4 without replacement) was carried out using a caliper of width 0.2 standard deviations of the logit model of the estimated score.

## Results

### Search Results and Characteristics of the Included Patients

**Figure 1:**
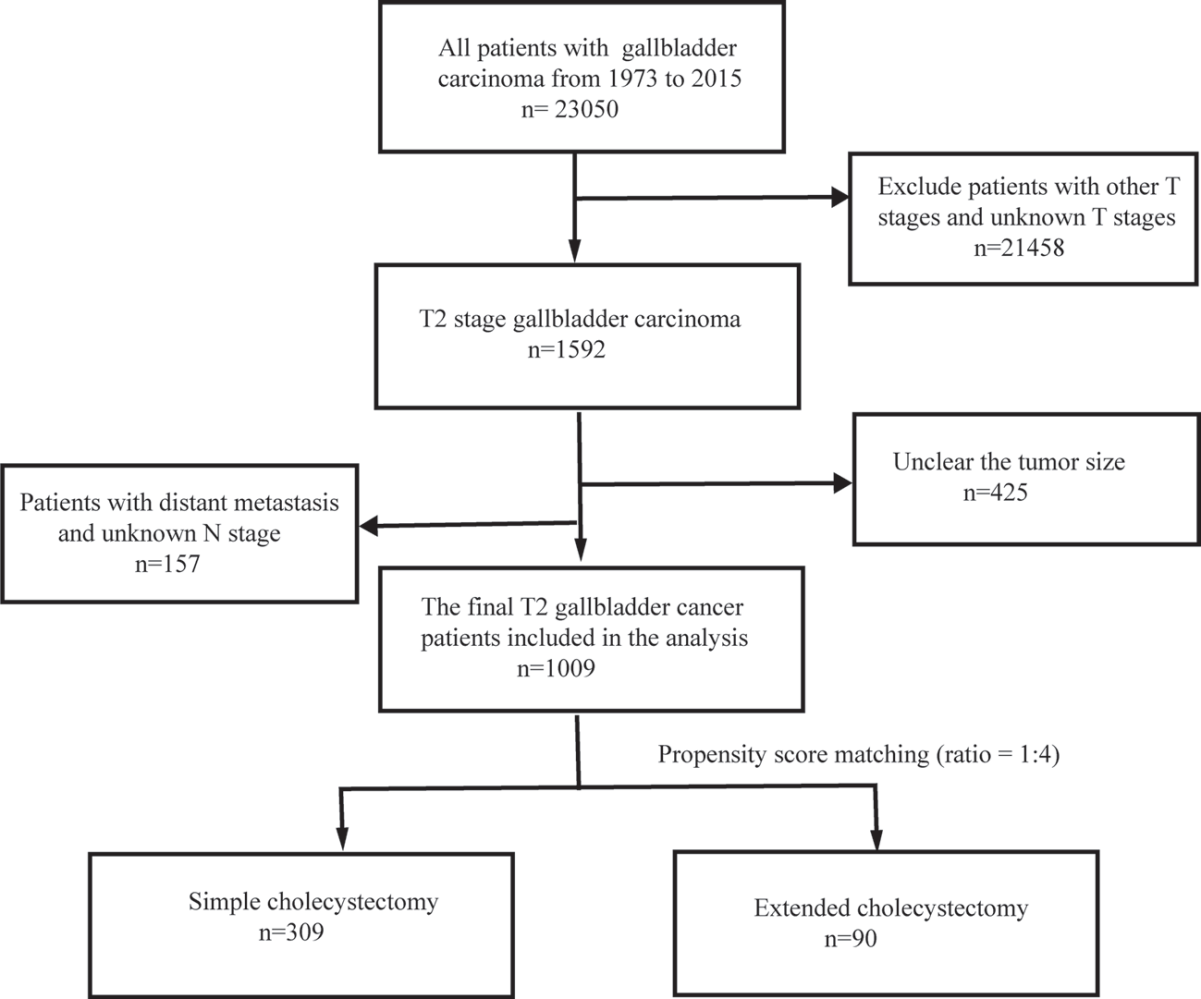
Flow chart for identification of the study population.

This retrospective study initially identified 1,592 patients with T2 GBC that were registered in the SEER database between 1973 and 2015 by applying the above criteria. However, 425 patients were excluded from further analysis due to unclear tumor size. In addition, further 157 patients were rejected due to the unclear distant metastasis and N-stage. Overall, 1,009 T2 GBC patients were enrolled in this study. The data screening process is shown in **Figure 1**.

### Population and Propensity Score Matching

Among the 1,009 eligible patients, 915 patients underwent a simple cholecystectomy and 94 patients received an extended cholecystectomy. Furthermore, 428 patients did not receive a regional lymphadenectomy, 372 patients received one to three regional lymphadenectomy, and 209 patients received four or more regional lymphadenectomy. The baseline clinicopathological characteristics are shown in **Table 1**. Patients who underwent extended cholecystectomy also received more region lymphadenectomy (p < 0.001). Overall, there were significant differences in clinicopathological features between the two groups of patients. Therefore, in order to balance the baseline characteristics, five unbalanced covariates (*i.e.*, grade, age at diagnosis, scope regional LN, N stage, and tumor size) were matched with propensity scores (ratio = 1:4). After pairing, a total of 399 patients were included with 309 patients in the simple cholecystectomy group, while 90 patients were part of the extended cholecystectomy group. There were no significant differences between the above-mentioned covariates, and the baseline characteristics between the two groups were balanced (**Table 2**).

**Table 1:** Patient baseline characteristics before propensity score matching.

Characteristics	Total (n)	Simple cholecystectomy	Extended cholecystectomy	P*
**Case**	1009	915	94	
**Race**				**0.01**
White	763	704	59	
Black	125	107	18	
Other	121	104	17	
**Age**		71.975 ± 12.4073	66.191 ± 12.4877	**<0.001**
**Sex**				0.905
Male	306	278	28	
Female	703	637	66	
**Clinical N staged**				**<0.001**
N1	751	698	53	
N2	248	210	38	
N3	10	7	3	
**Histological type (ICD-O-3)**				0.5
Adenocarcinoma		741	69	
Papillary or papillary adenocarcinoma		51	8	
Other				
**Differentiation**				0.373
Well differentiated	169	156	13	
Moderately differentiated	471	422	49	
Poorly differentiated	324	299	25	
Undifferentiated	20	17	3	
Unknown		21	4	
**Scope Reg LN**				**<0.001**
0	428	414	14	
1–3	372	339	33	
4+	209	162	47	
**Tumor size (mm)**		30 ± 20.73	37.31 ± 24.41	**0.001**
**TNM stage**				**<0.001**
II	751	698	53	
IIIB	248	210	38	
IVB	10	7	3	

Scope Reg LN, scope of regional lymphadenectomy; ICD-O-3, the 3rd edition of the International Classification of Diseases for Oncology; *-χ2 test or Fisher’s exact test, appropriately; the bold indicates statistical significance.

**Table 2:** Patient baseline characteristics after propensity score matching.

Characteristics	Total (n)	Simple cholecystectomy	Extended cholecystectomy	P^*^
**Case**	391	302	89	
**Race**				0.677
White	260	201	59	
Black	65	48	17	
Other	66	53	13	
**Age**		67.652 ± 12.176	67.022 ± 11.951	0.818
**Sex**				0.844
Male	111	85	26	
Female	280	217	63	
**Clinical N staged**				0.696
N1	236	185	51	
N2	149	113	36	
N3	6	4	2	
**Histological type (ICD-O-3)**				0.332
Adenocarcinoma				
Papillary or papillary adenocarcinoma				
Other				
**Differentiation**				0.828
Well differentiated	58	45	13	
Moderately differentiated	186	140	46	
Poorly differentiated	121	98	23	
Undifferentiated	10	7	3	
Unknown		12	4	
**Scope Reg LN**				0.521
0	67	53	14	
1–3	160	127	33	
4+	164	122	42	
**Tumor size (mm)**		32.841 ± 21.67	37.348 ± 24.860	0.348
**TNM stage**				0.696
II	236	185	51	
IIIB	149	113	36	
IVB	6	4	2	

Scope Reg LN, scope of regional lymphadenectomy; ICD-O-3, the 3rd edition of the International Classification of Diseases for Oncology; *-χ2 test or Fisher’s exact test, appropriately; the bold indicates statistical significance.

### Survival Outcome for Patients Received Simple Cholecystectomy or Extended Cholecystectomy, Regional Lymphadenectomy

**Figure 2:**
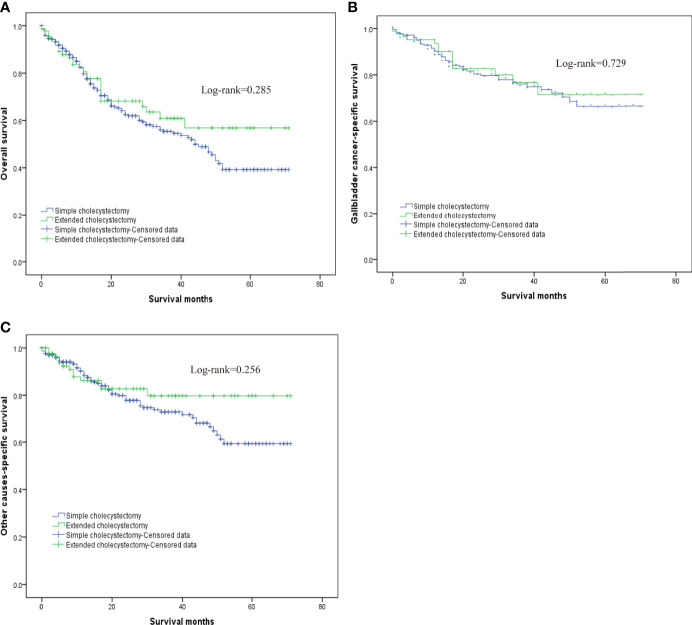
The overall survival for T2 gallbladder cancer comparison after propensity score matching between patients who underwent simple cholecystectomy and patients who underwent extended cholecystectomy. **(A)** Kaplan–Meier plots for overall survival. **(B)** Kaplan–Meier plots for cumulative gallbladder cancer-specific survival. **(C)** Kaplan–Meier plots for cumulative other cause-specific survival.

In order to evaluate the survival benefit of the scope of cholecystectomy, as well as the scope of regional lymphadenectomy, this study compared OS, G-CSS, and O-CSS after two types of surgery, with PSM eliminating the other unbalanced covariates. The OS at 1, 3, and 5 years in the extended cholecystectomy group *vs*. simple cholecystectomy group was 80.6 *vs*. 79.7%, 60.9 *vs*. 55.4%, 56.95 *vs*. 39.2%, respectively. In addition, the G-CSS at 1, 3, and 5 years in the extended cholecystectomy group compared to the simple cholecystectomy group was 93.5 *vs*. 90.2%, 76.7 *vs*. 76.0%, 71.6 *vs*. 66.4%, respectively. Finally, the 1-, 3-, and 5-year O-CSS in the extended cholecystectomy group *vs*. the simple cholecystectomy group was 86.3 *vs*. 88.4%, 79.8 *vs*. 73.0%, 79.8 *vs*. 59.3%, respectively. Furthermore, we also compared the OS, G-CSS, and O-CSS in patients with a different scope of lymphadenectomy by utilizing the log-rank (Mantel–Cox) test (**Figures 2A–C**). With regard to the OS, the extended cholecystectomy group did not show any significant improvements compared to the simple cholecystectomy group (**Figure 2**). The median OS was 45 *vs*. 48 months (p = 0.285). In addition, there were no significant differences in G-CSS between extended cholecystectomy and simple cholecystectomy alone (p = 0.729). For O-CSS, there were also no significant differences between the extended cholecystectomy and simple cholecystectomy alone (p = 0.256).

**Figure 3:**
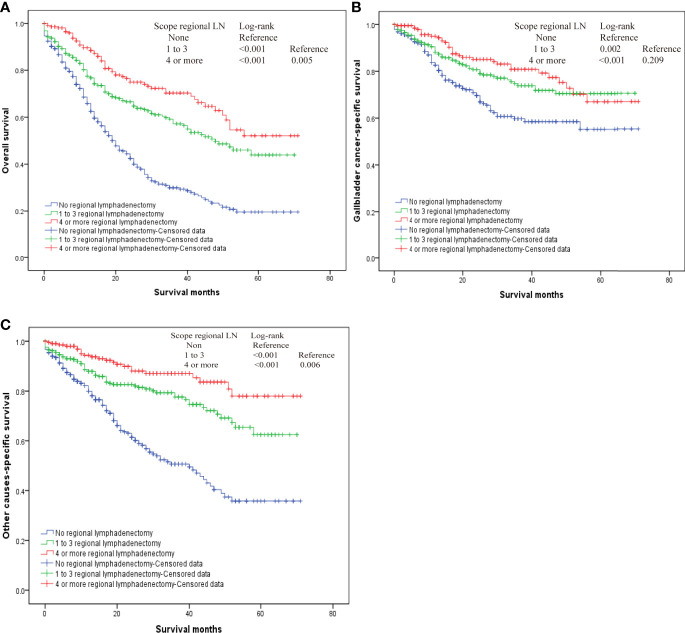
The overall survival for T2 gallbladder cancer who underwent regional lymphadenectomy. **(A)** Kaplan–Meier plots for overall survival. **(B)** Kaplan–Meier plots for cumulative gallbladder cancer-specific survival. **(C)** Kaplan–Meier plots for cumulative other cause-specific survival.

**Figure 4:**
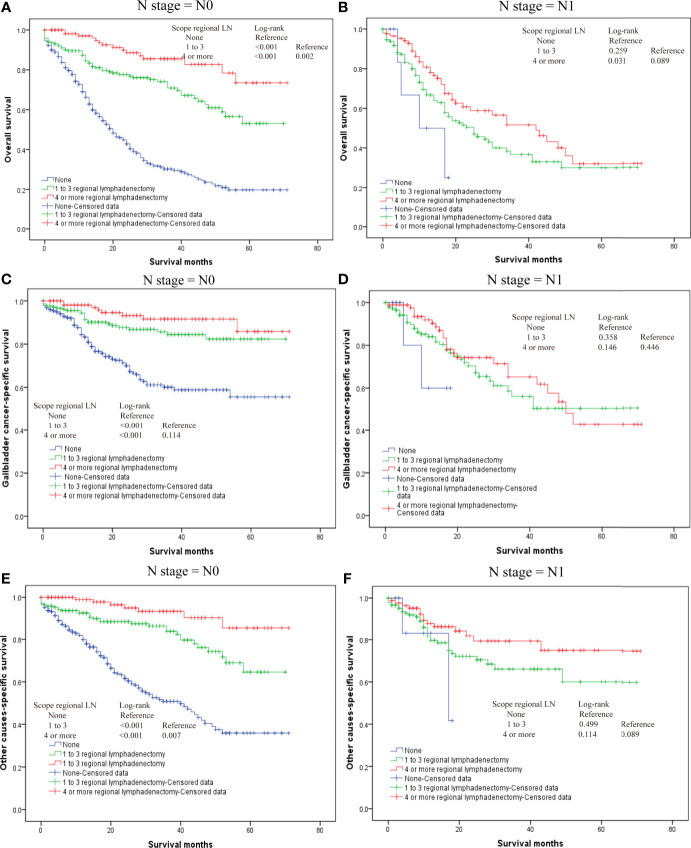
The overall survival for T2 gallbladder cancer in different N-stages who underwent regional lymphadenectomy. **(A)** Kaplan–Meier plots for overall survival in the N0 stage. **(B)** Kaplan–Meier plots for overall survival in N1 stage. **(C)** Kaplan–Meier plots for cumulative gallbladder cancer-specific survival in N0 stage. **(D)** Kaplan–Meier plots for cumulative gallbladder cause-specific survival in N1 stage. **(E)** Kaplan–Meier plots for cumulative other cause-specific survival in N0 stage. **(F)** Kaplan–Meier plots for cumulative other cause-specific survival in N1 stage.

Our results also demonstrated that regional lymphadenectomy likely benefits postoperative survival (**Figure 3**). Compared to no regional lymphadenectomy, patients that underwent regional lymphadenectomy had survival benefits with regard to the OS, G-CSS, and O-CSS (p < 0.05). However, with regard to OS and O-CSS, four or more regional lymphadenectomies were found to have a greater survival benefit compared to the one to three regional lymphadenectomies. Further, we discovered that the excision scopes of regional lymph nodes were significantly different in the groups with different N-stages (p < 0.001). Therefore, in order to eliminate the bias caused by the N-stage, we used the N-stage as a stratification factor to evaluate the impact of regional lymphadenectomy on prognosis of patients in different N-stages. In the N0 stage, regional lymphadenectomy was shown to bring improved survival benefits, and the extent of regional lymphadenectomy was shown to be positively correlated with survival benefit. On the contrary, most patients of N1 stage who underwent lymphadenectomy were found to have no significant differences in the survival between those who underwent one to three regional lymphadenectomies and those who received four or more regional lymphadenectomies. Since only few cases did not receive regional lymphadenectomy in the N1-group, we were not able to generate reliable conclusions about comparisons between patients who underwent regional lymphadenectomy and patients who did not receive regional lymphadenectomy in the N1-group. Although the log-rank test demonstrated significant differences, the probability of making type II errors was high, and no effective results were obtained. For the N2 stage, there were only 10 cases in total. Therefore, this study could not assess whether regional lymphadenectomy can bring significant benefits to patients in the N2 stage (**Figure 4**).

### Multivariate Regression Analysis and Nomogram for Survival Benefit Prediction

**Figure 5:**
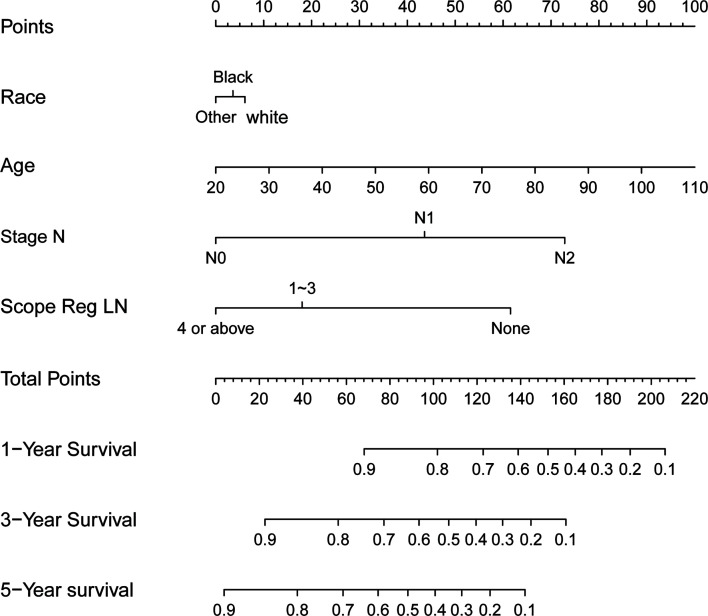
Nomograms for overall postoperative survival of T2 gallbladder cancer.

**Figure 6:**
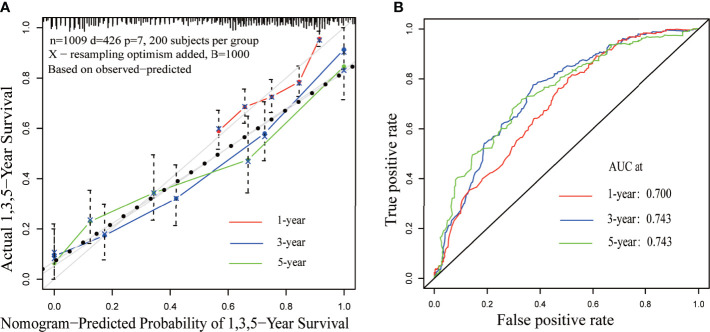
Calibration and discrimination validation. **(A)** The calibration curve demonstrates the comparison of the survival prediction of the model to the actual observed survival. **(B)** Receiver operating characteristic (ROC) curve internally validates the model performance for discrimination.

Results of the multiple regression model are shown in **Table 3**. The statistically significant covariates included age, scope of regional lymphadenectomy, N-stage, and tumor size. We utilized the *β-*coefficient of the model in order to develop a nomogram, and used internal verification to identify and calibrate the performance of the model (**Figure 5**). The calibration curve depicts good consistency between predicted survival and observed survival results (**Figure 6A**). We used area under the ROC curve to measure the ability to discriminate OS at 1, 3, and 5 years, which led to their area under curve (AUC) of 0.740, 0.743 and 0.743, respectively (**Figure 6B**).

**Table 3:** Univariate and multivariate Cox hazard analysis of risk factors for overall survival rate.

	Univariate analysis			Multivariate analysis		
	Hazard ratio	95% CI	P*	Hazard ratio	95% CI	P*
**Age (years)**	1.036	1.027–1.045	**<0.001**	1.024	1.015–1.034	**<0.001**
**Male gender**			0.205			0.278
Male	Reference			Reference		
Female	1.141	0.931–1.399	0.205	1.122	0.911–1.381	0.278
**Race**			0.162			0.540
White	Reference			Reference		
Black	1.273	0.907–1.787	0.163	1.21	0.857–1.709	0.279
Other	1.008	0.650–1.565	0.971	1.139	0.730–1.778	0.567
**Clinical N staged**			**0.029**			**<0.001**
N0	Reference			Reference		
N1	0.428	0.191–0.961	0.4	0.241	0.105–0.551	**0.001**
N2	0.524	0.23–1.192	0.123	0.575	0.249–1.329	0.195
**Differentiation**			**0.001**			**0.021**
Well differentiated; Grade I	Reference			Reference		
Moderately differentiated; Grade II	0.943	0.560–1.590	0.827	0.928	0.549–1.569	0.78
Poorly differentiated; Grade III	0.944	0.582–1.531	0.814	1.047	0.645–1.701	0.852
Undifferentiated; Grade IV/unknown	1.413	0.867–2.302	0.166	1.381	0.846,2.256	0.197
**Tumor size**	1.009	1.005–1.013	**<0.001**	1.008	1,003–1.012	**<0.001**
**Types of surgery**			0.058			0.798
Simple cholecystectomy	Reference			Reference		
Extended cholecystectomy	1.433	0.988–2.077	0.058	1.052	0.715–1.547	0.798
**Scope Reg LN**			**<0.001**			**<0.001**
0	Reference			Reference		
1–3	3.076	2.296–4.121	**<0.001**	3.846	2.699–5.479	**<0.001**
4+	1.556	1.138–2.127	**0.006**	1.516	1.097–2.094	**0.012**

Scope Reg LN, scope of regional lymphadenectomy; ICD-O-3, the 3rd edition of the International Classification of Diseases for Oncology; CI, confidence interval; *-χ2 test or Fisher’s exact test, appropriately; the bold indicates statistical significance.

## Discussion

Using a large database, such as SEER, and analyzing clinical data of more than 1,000 GBC patients can be very helpful for clinical practice. Herein, we show that, for T2 GBC, extended cholecystectomy does not provide improved survival than those undergoing simple cholecystectomy. However, for patients with negative lymph nodes, undergoing more regional lymphadenectomy provides significantly improved survival compared to no regional lymphadenectomy or fewer regional lymphadenectomy. In other words, for T2 N0 GBC patients, more regional lymphadenectomy is able to provide significant survival benefit, but undergoing extended cholecystectomy does not. This is one of the largest studies regarding the survival impact of surgery and regional lymphadenectomy among patients with T2 GBC and is consistent with previous studies which postulated that extended resection do not improve prognosis. For example, Kang et al. (4) reported a favorable survival of 75 cases without extended resection. Cho et al. (8) reported that, regardless of tumor location, the survival of T2 GBC patients who underwent hepatic resection was not better compared to patients without hepatic resection. Park et al. (7) conducted a similar study and concluded that extended hepatic resection for T2b GBC did not have an effect on long-term survival, and the number of regional lymphadenectomy is an important prognostic factor for T2 GBC.

At present, some guides and scholars (5, 9, 10) believe that the main purpose of hepatectomy for GBC is three-fold. First, it serves to prevent tumors from directly invading the liver from the gallbladder bed and achieve negative resection margin. Second, it helps prevent recurrence caused by micro-metastasis. Third, entire resection of the right liver Glisson’s sheath eliminates potential invasion of the hepatoduodenal ligament. Therefore, under condition of ensuring negative surgical margins, hepatic resection and extrahepatic bile duct resection may not be recommended in surgical treatment of pT2 GBC (3, 6).

One significant reason for the inconsistent conclusions among various studies on the resection of T2 GBC is that the scope of extended hepatic resection is inconsistent among those studies. The standard hepatic resection scope for T2 GBC has not yet been established. Despite the fact that some clinical guidelines recommend using IVb/V section resection or non-anatomical hepatectomy with a 2 cm rim around the edge of the gallbladder bed (9, 10), German guidelines recommend non-anatomical hepatectomy with a 3 cm rim around the gallbladder bed (5), while Korean guidelines recommend non-anatomical hepatectomy, with a margin of 2–3 cm (10). However, for GBC that directly invades the liver (classified as pT3 GBC), extended surgical resection is necessary, including hepatic resection, in order to achieve a radical resection. Extended hepatic resection and routine bile tube resection have not been shown to significantly improve prognosis of T2 GBC patients. Therefore, the decision to expand the resection should be based on standard of whether R0 resection is achieved. Extended hepatic resection or even pancreaticoduodenectomy should only be performed in selected patients due to high incidence of postoperative complications and surgical mortality (21). Some studies have reported that intrahepatic metastasis is more common in T2b-GBC patients, which is closely related to direct drainage of lymph nodes into the liver (11, 12). Cho et al. demonstrated that T2b GBC is more prone to intrahepatic recurrence than T2a GBC; however, recurrence of T2b GBCs is systemic, and as discussed previously, there is no evidence that partial hepatectomy is able to prevent postoperative liver metastasis (3, 6, 7).

Herein, we found that lymphadenectomy plays a significant role in the radical surgical treatment of T2 GBC, particularly among patients with negative regional lymph nodes (N0 stage). Lymphadenectomy can reduce the incidence of death due to GBC, as well as to other causes. As the scope of lymphadenectomy extends, risk of other cause of death also becomes significantly reduced. However, patients with positive regional lymph nodes do not receive benefit from more regional lymphadenectomy. From the multivariate Cox hazard model, it can be seen that survival of patients with positive lymph nodes (N1 stage) is about 25% of those with negative lymph nodes (N0). Positive lymph node indicates that the tumor cells have distantly metastasized. Furthermore, surgical treatment is unable to eliminate hidden metastasis in the distance. Therefore, T2 patients with positive lymph nodes need to undergo chemotherapy and radiotherapy. Studies have reported that adjuvant chemotherapy can significantly improve survival of patients with lymph node metastasis (13, 14). Regardless of whether it is simple cholecystectomy or extended cholecystectomy, achievement of R0 excision and wider range of lymph node excision are the fundamental purpose of surgical treatment. Therefore, we believe that a multidisciplinary and integrated approach to GBC can likely benefit GBC patients.

In recent years, increasingly more progress has been made in survival prediction models and prediction tools. The risk factors and prognostic prediction models of many cancers have been explored, including stomach cancer, colorectum cancer, and breast cancer. Although predictive models cannot replace evidence from prospective randomized clinical trials with large samples, these tools are particularly helpful when making clinical decisions as clinical trial data are scarce. In this study, we developed a nomogram model using available data from the SEER database to assess survival benefits associated with cholecystectomy, regional lymphadenectomy, as well as other risk factors. By considering these important clinical factors and survival of patients that underwent different treatment strategies, this model has certain reference value in clinical practice.

## Conclusion

There were no significant postoperative survival differences between simple cholecystectomy and extended cholecystectomy for treatment of T2 GBC. Regional lymphadenectomy is able bring significant survival benefits to T2 GBC patients with negative lymph nodes, and the greater the scope of lymphadenectomy, the greater the benefit. However, regional lymphadenectomy does not seem to benefit survival of lymph node-positive patient. Thus, this still requires further validation from high-quality large-sample prospective studies.

## Limitation

The study has several limitations. First, although this study performed a propensity score matching based on patient baseline characteristics in order to minimize bias, some unavailable confusing factors may generate residual bias. Second, although SEER data were obtained through professionally trained professionals, its retrospective nature makes it highly likely that the data were exposed to selection bias. Third, the databases are maintained by large medical institute that have little control over the local practices, which limit the current analysis to the available variables in the database.

## Data Availability Statement

The raw data supporting the conclusions of this article will be made available by the authors, without undue reservation.

## Author Contributions 

WZ, the first author, was responsible for drafting the manuscript, as well as the acquisition, analysis and interpretation of data. ZH, W-eW and XC contributed to the conception and design of the current study. All authors contributed to the article and approved the submitted version.

## Funding

This work was supported by Sanming Project of Medicine in Shenzhen (No. SZSM202011010).

## Conflict of Interest

The authors declare that the research was conducted in the absence of any commercial or financial relationships that could be construed as a potential conflict of interest.

## Publisher’s Note

All claims expressed in this article are solely those of the authors and do not necessarily represent those of their affiliated organizations, or those of the publisher, the editors and the reviewers. Any product that may be evaluated in this article, or claim that may be made by its manufacturer, is not guaranteed or endorsed by the publisher.
